# Optimizing harvest of corn stover fractions based on overall sugar yields following ammonia fiber expansion pretreatment and enzymatic hydrolysis

**DOI:** 10.1186/1754-6834-2-29

**Published:** 2009-11-24

**Authors:** Rebecca J Garlock, Shishir PS Chundawat, Venkatesh Balan, Bruce E Dale

**Affiliations:** 1 Biomass Conversion Research Laboratory, Department of Chemical Engineering and Materials Science, Michigan State University, 3900 Collins Road, Lansing, MI 48910-8596, USA

## Abstract

**Background:**

Corn stover composition changes considerably throughout the growing season and also varies between the various fractions of the plant. These differences can impact optimal pretreatment conditions, enzymatic digestibility and maximum achievable sugar yields in the process of converting lignocellulosics to ethanol. The goal of this project was to determine which combination of corn stover fractions provides the most benefit to the biorefinery in terms of sugar yields and to determine the preferential order in which fractions should be harvested. Ammonia fiber expansion (AFEX) pretreatment, followed by enzymatic hydrolysis, was performed on early and late harvest corn stover fractions (stem, leaf, husk and cob). Sugar yields were used to optimize scenarios for the selective harvest of corn stover assuming 70% or 30% collection of the total available stover.

**Results:**

The optimal AFEX conditions for all stover fractions, regardless of harvest period, were: 1.5 (g NH_3 _g^-1 ^biomass); 60% moisture content (dry-weight basis; dwb), 90°C and 5 min residence time. Enzymatic hydrolysis was conducted using cellulase, β-glucosidase, and xylanase at 31.3, 41.3, and 3.1 mg g^-1 ^glucan, respectively. The optimal harvest order for selectively harvested corn stover (SHCS) was husk > leaf > stem > cob. This harvest scenario, combined with optimal AFEX pretreatment conditions, gave a theoretical ethanol yield of 2051 L ha^-1 ^and 912 L ha^-1 ^for 70% and 30% corn stover collection, respectively.

**Conclusion:**

Changing the proportion of stover fractions collected had a smaller impact on theoretical ethanol yields (29 - 141 L ha^-1^) compared to the effect of altering pretreatment and enzymatic hydrolysis conditions (150 - 462 L ha^-1^) or harvesting less stover (852 - 1139 L ha^-1^). Resources may be more effectively spent on improving sustainable harvesting, thereby increasing potential ethanol yields per hectare harvested, and optimizing biomass processing rather than focusing on the selective harvest of specific corn stover fractions.

## Background

Corn stover, the aboveground, vegetative portion of maize (*Zea mays *L.), makes up roughly 80% of all agricultural residues produced in the USA [1]. Data on annual corn stover production in the USA are not readily available, so various sources have independently estimated that anywhere from 200 to 250 million dry tons of corn stover are produced per year [1-4]. Sustainably harvested corn stover could be used as a feedstock for a variety of applications, including lignocellulosic ethanol production. It has been estimated that 38.4 billion liters of ethanol per year could be produced from North American corn stover, assuming that 40% of the stover is collected [5]. It is widely acknowledged that a percentage of the produced corn stover should be retained on the field following harvest in order to prevent soil erosion and maintain soil organic carbon (SOC) levels. The amount that can be sustainably harvested is highly debated and depends heavily on cropping practices, climate, topography and soil type [4,6-8]. Estimates on the amount of corn stover that can be sustainably harvested vary widely because of these factors, anywhere from 20-80% [1,5,6].

Lignocellulosic feedstocks, such as corn stover, derive their name from the three primary components of the plant cell wall: cellulose, hemicellulose and lignin. The complex polysaccharides, cellulose and hemicellulose, must be broken down into monomeric form (primarily glucose and xylose) prior to microbial fermentation into ethanol or other valuable products. High sugar yields require a two-step process: generally a chemical and/or physical pretreatment step followed by enzymatic hydrolysis of the polysaccharides. Previous work has shown that ammonia fiber expansion (AFEX) is a promising pretreatment that can be used in the process of converting corn stover polysaccharides into ethanol as a liquid fuel source [9-12]. AFEX pretreatment uses concentrated ammonia-water mixtures under moderate temperatures (60°-180°C) and high pressures (200-1000 psi) to disrupt the cellular structure of the plant material by decrystallizing the cellulose, partially depolymerizing and solubilizing the hemicellulose and altering the form, location and structure of lignin [9,11].

The structure and composition of the plant cell wall depends on a number of factors including: developmental stage at harvest, geographical origin, type of tissue and other external factors including season of harvest and environmental conditions experienced during growth [13]. Corn stover, like most grasses, experiences considerable compositional changes throughout the yearly growth period as well as significant variation between the various fractions of the plant (that is, leaf versus stem) [14-16]. Largely because of these differences in composition, stover fractions have been shown to respond differently to pretreatment and enzymatic hydrolysis, resulting in different sugar yields [17-19]. It is reasonable to assume that differences in composition, due largely to differences in morphology and cell and tissue organization, could cause different stover fractions to have different optimal pretreatment conditions for maximizing sugar yields. For example, wheat straw leaves, when treated with dilute NaOH, required less severe pretreatment conditions to optimize glucan yields than stem internodes and nodes [20]. The same might be true for corn stover pretreated with ammonia (or AFEX). Maximum sugar yields from individual fractions would be one criterion for determining which fractions should be left on the field following harvest. Assuming that there are no other constraining factors, it would be most logical to harvest the least recalcitrant biomass and leave the remainder for erosion control and soil organic carbon maintenance [21]. Crofcheck and Montross recommended, based on glucose yields from fractionated corn stover, a roughly 30% corn stover harvest scenario where the selectively harvested corn stover (SHCS) was composed of all of the available cobs and 74% of the leaves and husks, leaving the most recalcitrant stalks on the field [17].

For our experiment, AFEX followed by enzymatic hydrolysis was performed on four different corn stover fractions (stem, leaf, husk and cob) from September (early) and November (late) harvests. The objectives of this project were: (1) to determine whether individual stover fractions have different optimal AFEX conditions and whether this is different from previously optimized values for homogeneously milled corn stover [9,11]; (2) to discover which fractions give the highest glucose and xylose yields at optimal pretreatment conditions; and (3) to model optimal harvest scenarios, assuming 30% and 70% collection of total available dry corn stover, based on the maximum monomeric glucose and xylose yields from each fraction.

## Results

### Composition analysis

The composition of each of the corn stover fractions from each harvest is listed in Table 1. The value of the 'other' column was determined by the difference of the total of the other columns from 100%. The standard deviation is representative of three replicates. Statistically, the early and late stem and the late leaves and husk had the highest glucan content, while the early leaves and late cob had the lowest glucan content. The xylan content of the late fractions was significantly higher than their early counterparts and tended to decrease from late cob > late husk > late stem > late leaves > early stem > early leaves. The acid-insoluble lignin content was similar for all fractions, except for the cob, which had the highest lignin content, and the late husk, which had statistically less lignin than the late stem. The ash content of all fractions were statistically different and decreased from early leaves > late leaves > early stem > late stem > late husk > late cob.

**Table 1:** Corn stover composition for early and late harvest stover fractions.

		Corn stover fraction composition (% dry biomass)				
Corn stover fraction		Glucan	Xylan	Acid-insoluble lignin	Ash	Other
**Early**	**Leaves**	27.5^b ^± 3.2	17.8^e ^± 1.7	13.2^bc ^± 0.7	7.3^a ^± 0.13	34.2
	**Stem**	35.1^a ^± 2.6	19.0^de ^± 1.1	14.9^bc ^± 0.2	3.4^c ^± 0.10	27.6

**Late**	**Leaves**	35.3^a ^± 1.2	21.8^cd ^± 0.6	13.6^bc ^± 1.7	6.0^b ^± 0.25	23.3
	**Stem**	37.8^a ^± 0.9	23.6^bc ^± 0.4	16.9^b ^± 0.5	2.4^d ^± 0.08	19.3
	**Husk**	39.0^a ^± 2.2	26.5^b ^± 1.5	11.6^c ^± 0.3	2.1^e ^± 0.11	20.8
	**Cob**	27.5^b ^± 1.1	32.3^a ^± 1.3	25.8^a ^± 2.6	1.1^f ^± 0.02	13.3

Values with different superscripts in an individual column were statistically different using Tukey's pairwise comparison with α = 0.05. [The 'other' column determined by difference from 100%.]

### AFEX pretreatment and hydrolysis

Pretreatment conditions for AFEX-treated corn stover have been previously optimized at 1.0 (g NH_3 _g^-1 ^dry biomass), 60% moisture content (dry-weight basis; dwb), 90°C and 5 min residence time [9,11]. These conditions were treated as the 'base case' for the analysis of pretreatment conditions. The effect of pretreatment conditions on monomeric glucose and xylose yields following hydrolysis, was tested by varying one process parameter (temperature, ammonia loading, moisture content or residence time) at a time (for example, raising the temperature from 90°C to 100°C). Once the preliminary data had been gathered, the untreated control, base case and best case were supplemented with xylanase during hydrolysis to observe the effect on sugar yields.

**Figure 1:**
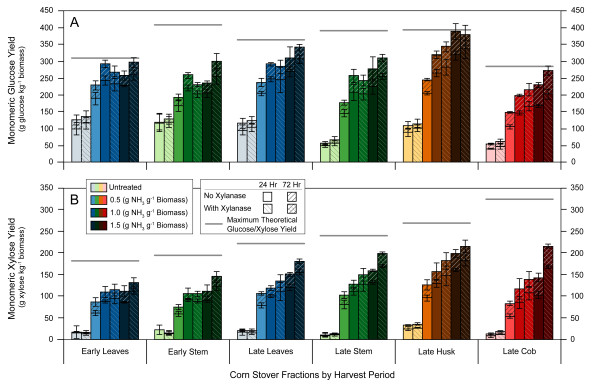
**Effect of ammonia fiber expansion (AFEX) pretreatment ammonia loading and xylanase addition on enzymatic hydrolysis monomeric sugar yields**. Glucose yields are reported in part A and xylose yields are in part B. All AFEX runs were kept at constant moisture content (60% dry-weight basis), temperature (90°C) and residence time (5 min). Yields are in terms of sugar available in untreated dry biomass.

Figure 1 shows the monomeric glucose and xylose yields for a variety of conditions with particular comparisons between untreated and AFEX treated materials at a range of ammonia loadings. The effect of xylanase addition to the enzyme cocktail can also be observed in Figure 1. Error bars in all figures represent the mean ± 1 standard deviation. From Figure 1, it can be seen that AFEX substantially improves both glucose and xylose monomeric sugar yields for all harvest periods and corn stover fractions when compared to untreated materials.

The increase in ammonia loading from 0.5 to 1.5 (g NH_3 _g^-1 ^biomass) had different effects on early harvest and late harvest corn stover fractions. For the early harvest stover without xylanase addition, glucose yields peak at 1.0 (g NH_3 _g^-1 ^biomass). This optimum is similar to what has been seen previously with AFEX-treated corn stover [9,11], which may indicate that that material was from an earlier harvest. The xylose yields are relatively unaffected by any further increase above 1.0 (g NH_3 _g^-1 ^biomass). However, when performing the same experiment with the late harvest corn stover, there is an increase in both glucose and xylose yields for all fractions when increasing from 1.0 to 1.5 (g NH_3 _g^-1 ^biomass).

Xylanase addition had little to no effect on the increase of either glucose or xylose sugar yields in untreated corn stover fractions. For AFEX-treated early harvest fractions, the addition of xylanase at 1.0 (g NH_3 _g^-1 ^biomass) had no effect on monomeric xylose yields and it slightly lowered glucose yields. At 1.5 (g NH_3 _g^-1 ^biomass), all fractions and harvests experienced an increase in both the monomeric xylose and glucose yields with the addition of xylanase.

The leaf and stem, for both early and late harvests, have similar glucose yields at 1.5 (g NH_3 _g^-1 ^biomass) ammonia loading. However, the leaf glucan is more digestible, as seen by the greater yield (percent of maximum theoretical glucan available). The late harvest husk approaches theoretical glucose yields at the optimal condition of 1.5 (g NH_3 _g^-1 ^biomass). As a result of this, the addition of xylanase for this pretreatment condition increases husk xylose yields slightly but not the glucose yields, as is seen in the other fractions. With the addition of xylanase at 1.5 (g NH_3 _g^-1 ^biomass), the cob and leaf also approach near theoretical glucose yields.

**Figure 2:**
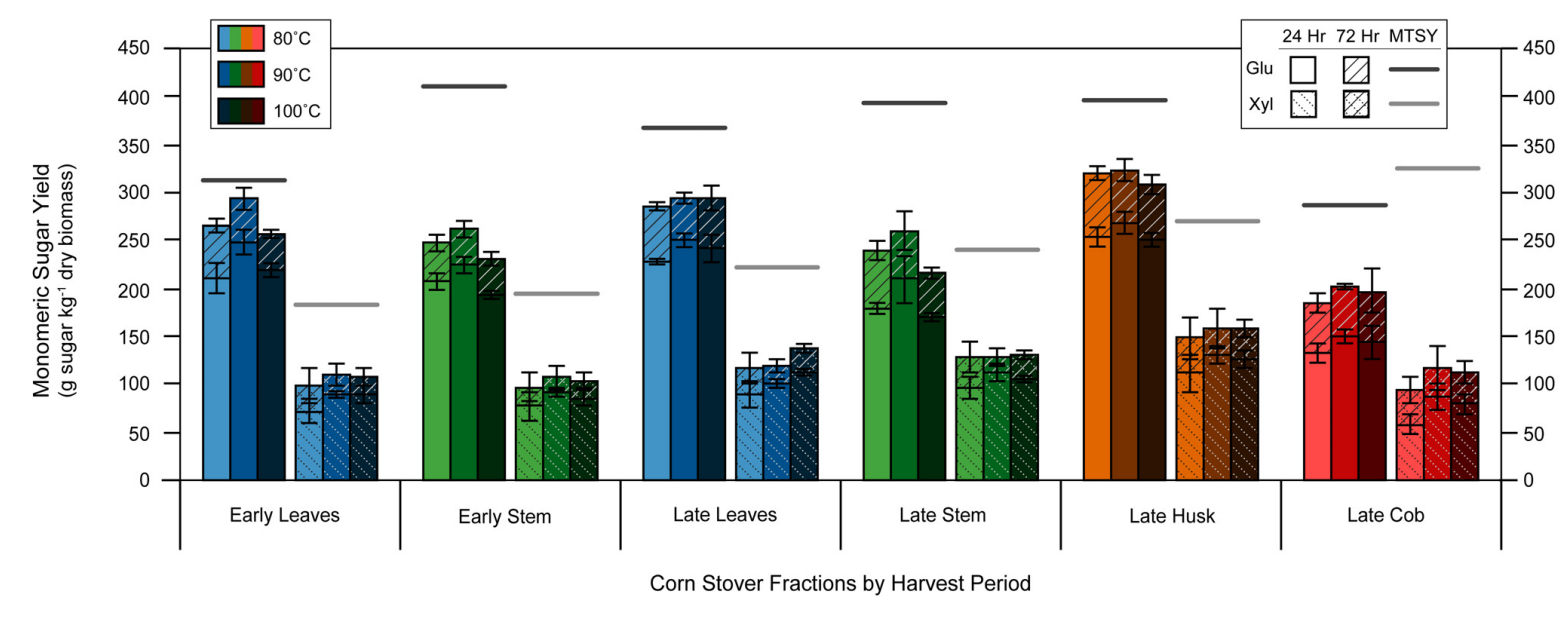
**Effect of ammonia fiber expansion (AFEX) pretreatment temperature on enzymatic hydrolysis monomeric sugar yields**. All AFEX runs were kept at a constant moisture content (60% dry-weight basis), ammonia loading (1.0 g NH_3 _g^-1 ^dry biomass) and residence time (5 min). Yields are in terms of sugar available in untreated dry biomass. Glu = glucose, Xyl = xylose, MTSY = maximum theoretical sugar yield.

Figure 2 shows the effect of pretreatment temperature on glucose and xylose yields from corn stover fractions. Altering the temperature by 10°C from the base case had little effect on glucose and xylose yields. There is a definite peak in glucose yields at 90°C for the early harvest but the late harvest has no apparent difference in yields for 80°, 90° or 100°C. In a previous work [9], raising the temperature above 90°C had a negative impact on ethanol yields from simultaneous saccharification and fermentation.

**Figure 3:**
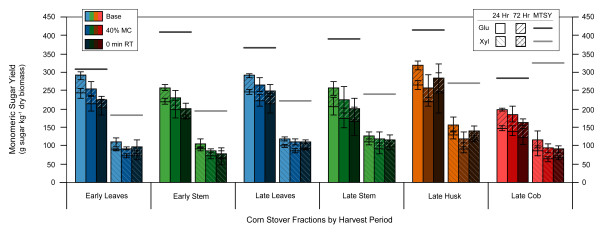
**Effect of ammonia fiber expansion (AFEX) moisture content and residence time on enzymatic hydrolysis monomeric sugar yields**. Base AFEX conditions: moisture content (60% dry-weight basis), ammonia loading (1.0 g NH_3 _g^-1 ^dry biomass), temperature (90°C) and residence time (5 min). Yields are in terms of sugar available in untreated dry biomass. MC = moisture content, RT = residence time, Glu = glucose, Xyl = xylose, MTSY = maximum theoretical sugar yield.

Decreasing the moisture content to 40% (dwb) and eliminating the residence time (the time for which the reactor was held at the set temperature following heat-up) each had a negative impact on glucose and xylose yields for all fractions (Figure 3). For all stover fractions, except the late husk, it was more detrimental in terms of sugar yields to decrease the residence time rather than the moisture content.

### Statistical analysis

**Figure 4:**
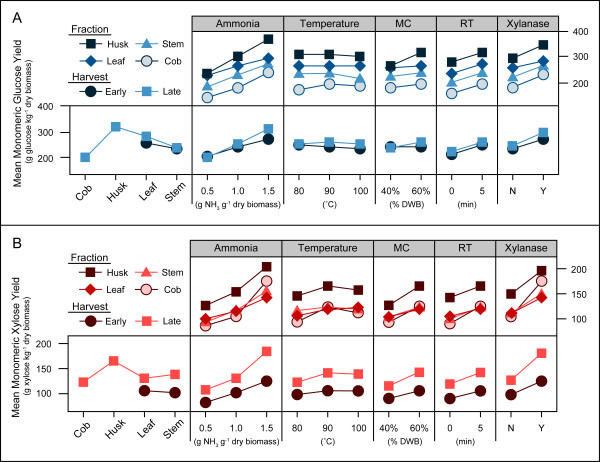
**Interaction effect plot of ammonia fiber expansion parameters, stover fraction and harvest period on monomeric sugar yields**. Glucose yields are reported in part A and xylose yields are reported in part B, both of which are in terms of untreated dry biomass following 72 h enzymatic hydrolysis. MC = moisture content, RT = residence time, DWB = dry-weight basis, N = no xylanase added, Y = xylanase added (10% of total cellulase protein).

Multivariate analysis of variance (MANOVA) was conducted in order to determine the significance of harvest date, corn stover fraction, AFEX parameters and xylanase addition on both the 24 hour and 72 hour monomeric glucose and xylose yields. Interactive effects were also examined between harvest date and stover fraction and each of the other parameters. As the conclusions regarding significance were the same for 24 hour and 72 hour yields for both glucose and xylose (Table 2), only the 72 hour yields were used for the interactive effects plot (Figure 4).

**Table 2:** Analysis of variance for factors influencing sugar yields.

	*P*-value			
Factor	24 h Glucose	72 h Glucose	24 h Xylose	72 h Xylose
Harvest date	0.775	0.437	0.000*	0.000*
Corn stover fraction	0.000*	0.006*	0.526	0.528
Ammonia loading	0.000*	0.000*	0.000*	0.000*
Temperature	0.082	0.161	0.000*	0.022*
Moisture content	0.018*	0.002*	0.000*	0.000*
Residence time	0.001*	0.000*	0.003*	0.000*
Xylanase addition	0.001*	0.002*	0.000*	0.000*
Harvest × ammonia	0.007*	0.001*	0.001*	0.002*
Harvest × temperature	0.918	0.932	0.824	0.392
Harvest × moisture	0.687	0.762	0.943	0.424
Harvest × residence time	0.829	0.719	0.377	0.317
Harvest × xylanase	0.919	0.760	0.111	0.063
Fraction × ammonia	0.288	0.080	0.416	0.152
Fraction × temperature	0.746	0.684	0.588	0.400
Fraction × moisture	0.278	0.075	0.163	0.109
Fraction × residence time	0.916	0.859	0.715	0.542
Fraction × xylanase	0.711	0.300	0.008*	0.030*

*Significant at α = 0.05.

Glucose yields were significantly affected by three of the AFEX pretreatment conditions: ammonia loading, moisture content and residence time, but not by temperature. Glucose yields were also dependent on the corn stover fraction and whether xylanase was added to the hydrolysis cocktail. Of the interactive effects analysed, only harvest date × ammonia loading had any significant affect on monomeric glucose yields. If the α-value is increased to 0.1, the fraction × ammonia and fraction × moisture also significantly affect 72-hour glucose yields. However, compared to the majority of the other significant parameters (except the moisture content and harvest × ammonia effect on 24-hour glucose yields), which are significant at α < 0.005, the effect of these two interactions on the glucose yield seems minimal.

Xylose yields were significantly affected by all four AFEX pretreatment conditions, including temperature. Unlike the case for glucose yields, xylose yields were not significantly affected by corn stover fraction but they were affected by both the harvest date and the addition of xylanase to the hydrolysis cocktail. There were also interactive effects on xylose yields from harvest date × ammonia loading and corn stover fraction × xylanase addition.

When analyzing the interactive effects plot, significant interactive effects will have very different slopes for the different lines in that portion of the graph. For example, when observing the interactive effect of harvest × ammonia on xylose yields, the slope of the early and late harvest lines are roughly the same when the ammonia loading is increased from 0.5 to 1.0 (g NH_3 _g^-1 ^biomass). However, when the ammonia loading is increased from 1.0 to 1.5 (g NH_3 _g^-1 ^biomass), the slope of the late harvest line is significantly steeper than the slope of the early harvest line. This difference in slope signifies that most of the impact of ammonia loading on this interaction is due to the second, not the first increase. This implies that the higher ammonia loading has a greater effect on the late harvest than the early harvest.

### Optimization of harvest scenarios

**Figure 5:**
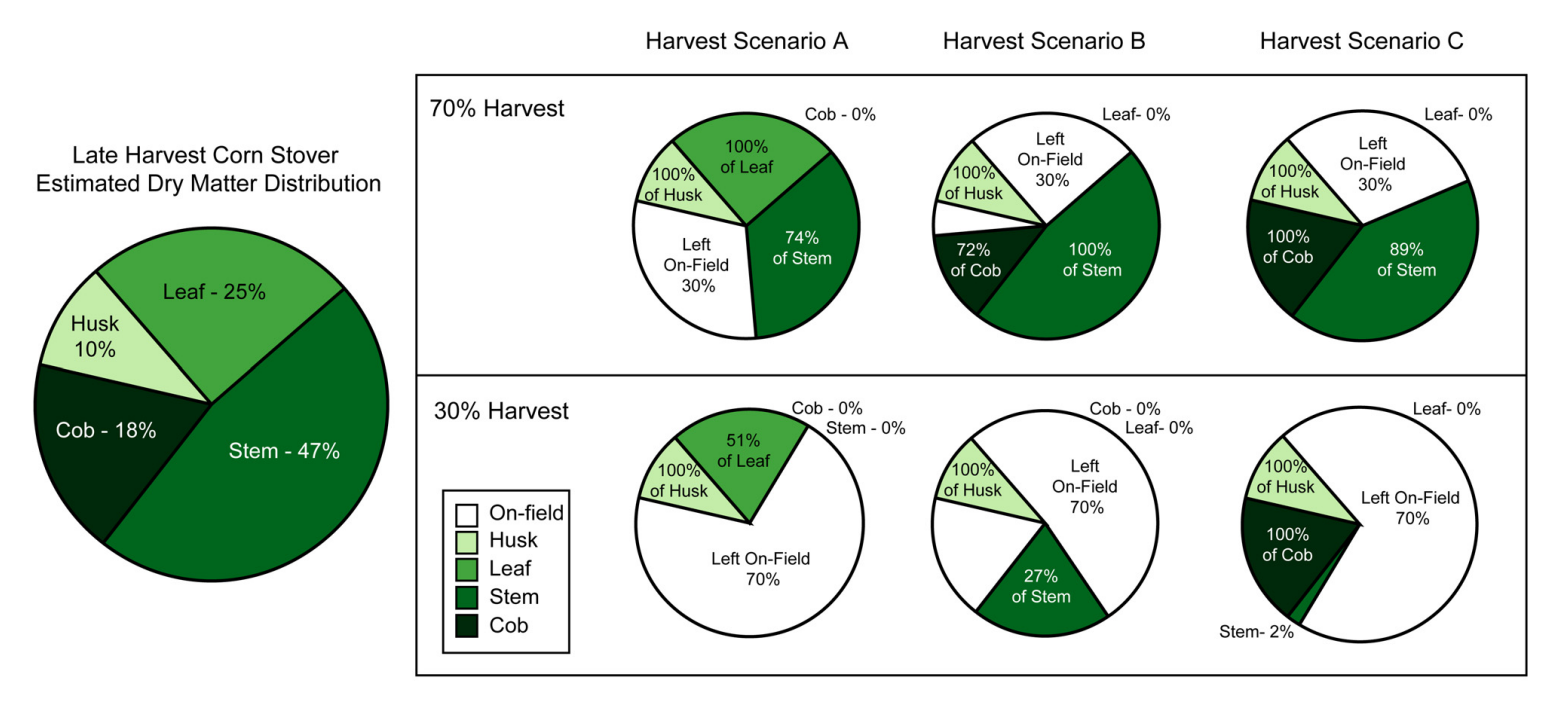
**Estimated dry matter distribution for 70% and 30% (dry-weight basis) harvest of late harvest corn stover**. Percentages of the individual fractions harvested are based on the total amount of each fraction available.

The conditions selected resulted in three scenarios for selectively harvesting corn stover (Table 3) because the harvest scenario to maximize glucose yields was the same for both ammonia loadings. The relative amounts of harvested fractions for each scenario are represented in Figure 5 for both the 70% and 30% harvests. A comparison of Tables 4 and 5 reveals that the amount of corn stover harvested has the largest impact on theoretical ethanol yield per hectare. Decreasing stover collection from 70% of available material to 30%, with the same harvest scenario, decreased theoretical ethanol yields by 852 - 1139 L ha^-1^. Decreasing the ammonia loading from 1.5 to 1.0 (g ammonia g^-1 ^biomass) for the same harvest scenario caused a decrease in the theoretical ethanol yield of 150 - 462 L ha^-1^, while switching desired sugars from glucose to xylose (that is, changing harvest scenarios but keeping stover collection and AFEX and enzymatic hydrolysis conditions constant) caused a decrease in the theoretical ethanol yield of 29 - 64 L ha^-1^. In order to determine the sensitivity of changing the harvest scenario, the model was also run assuming the worst case scenario, where the biomass was harvested in a manner that would give the worst possible sugar yields. The worst case scenario led to a decrease in the theoretical ethanol yields per hectare ranging from 81 - 141 L ha^-1^. As expected, when comparing untreated corn stover to the AFEX-treated cases (data not shown), the theoretical ethanol yield was substantially lower for the untreated cases: a decrease of 1234 - 1695 L ha^-1 ^for the 70% harvest and 527 - 719 L ha^-1 ^for the 30% harvest.

**Table 3:** Optimized harvest scenarios based on desired sugar and ammonia fiber expansion ammonia loading.

Harvest scenario	A	B	C
Optimized sugar	Glucose/total	Xylose	Xylose
Ammonia loading **(g NH**_3_**g**^-1 ^**dry SHCS)**	1.0, 1.5	1.0	1.5
**Best fraction**	Husk	Husk	Cob
	Leaf	Stem	Husk
	Stem	Cob	Stem
**Worst fraction**	Cob	Leaf	Leaf

SHCC, selectively harvested corn stover

**Table 4:** Estimated yields for 70% collection of selectively harvested corn stover (SHCS) following ammonia fiber expansion, enzymatic hydrolysis and fermentation.

		1.0 g NH_3 _g^-1 ^dry SHCS			1.5 g NH_3 _g^-1 ^dry SHCS		
Yield		Harvest scenario A	Harvest scenario B	Worst Case scenario	Harvest scenario A	Harvest scenario C	Worst case scenario
g sugar kg^-1 ^dry SHCS	**Glucose**	273.7	254.2	240.9	331.5	310.8	303.1
	**Xylose**	150.0	153.1	146.6	195.7	206.8	203.2
	**Total**	423.6	407.3	387.5	527.2	517.5	506.3

L kg^-1 ^dry SHCS	**Theoretical ethanol**	0.274	0.263	0.250	0.341	0.335	0.327

L ha^-1^	**Theoretical ethanol**	1648	1585	1508	2051	2014	1970

**Table 5:** Estimated yields for 30% collection of selectively harvested corn stover (SHCS) following ammonia fiber expansion, enzymatic hydrolysis and fermentation.

		1.0 g NH_3 _g^-1 ^dry SHCS			1.5 g NH_3 _g^-1 ^dry SHCS		
Yield		Harvest scenario A	Harvest scenario B	Worst case scenario	Harvest scenario A	Harvest scenario C	Worst case scenario
g sugar kg^-1 ^dry SHCS	**Glucose**	305.1	278.2	228.7	354.4	311.3	288.2
	**Xylose**	151.8	161.3	144.0	192.7	215.4	210.2
	**Total**	456.9	439.5	372.6	547.1	526.7	498.3

L kg^-1 ^dry SHCS	**Theoretical ethanol**	0.295	0.284	0.241	0.354	0.340	0.322

L ha^-1^	**Theoretical ethanol**	762	733	621	912	878	831

## Discussion

### Composition analysis

Fractions from the late harvest tended to have a slightly higher percentage of cell wall components (although not always significant) and slightly lower percentage of ash compared to their early harvest counterparts. For corn stover, the increase in lignin and cellulose and the decrease in ash have been observed elsewhere [22,23]. There is also a general increase in all cell wall components with a decrease in soluble solids and non-structural carbohydrates and an increase in lignin and xylan with increasing maturity [15,24]. This observed increase in the cellulose (glucan), hemicellulose (glucan and xylan) and lignin content is due to the secondary thickening of the plant cell wall that continues to occur for as long as the plant matures. During this time there is also a decrease in ash content [14]. However, while there is a continual change in the dry matter composition until late in the season, there tend to be very small changes during the grain harvest period [2,15], the time during which our samples were harvested.

### AFEX pretreatment

Based on the final total sugar yields, the optimal AFEX pretreatment conditions were observed to be consistent for all fractions, for both early and late harvest corn stover: 1.5:1 (g NH_3 _g^-1 ^biomass), 60% moisture content (dwb), 90°C, 5 min residence time and 10% xylanase addition (mg xylanase protein mg^-1 ^cellulase protein), in addition to the standard enzyme mixture used during enzymatic hydrolysis.

For AFEX-treated early harvest fractions, the addition of xylanase at 1.0 (g NH_3 _g^-1 ^biomass) had no effect on monomeric xylose yields and slightly lowered the glucose yields. This drop in glucose yields could be due to the competition for binding sites on the cellulose chains between enzymes in the xylanase and cellulase mixtures. The fact that there is no increase in xylose yields with the addition of xylanase supports this conclusion. If the xylanase, which has a much lower cellulase activity [25], is competitively binding to the cellulose instead of the xylan, this could result in a decrease in glucose yields with no significant change in xylose yields.

The higher optimal ammonia loading for the late harvest fractions compared with the early harvest could be due to a number of reasons. AFEX, by the ammoniation of the active methoxyl sites of lignin [26], may be preventing the lignin from binding to the hydrolysis enzymes. This may be one of the main reasons for the increase of 0.5 to 1.0 (g NH_3 _g^-1 ^biomass). However, if this were the reason for the difference in optimum ammonia loading between the early and late harvests, then the lignin content of the later harvest should be greater. This is not the case, however, as statistically the lignin contents of the early and late fractions are identical. The difference in optimal ammonia loading is more likely to be due to the increase in xylan content and possibly the increased cross-linking between hemicellulose and lignin from the early to late harvest. Ferulate cross-linking occurs between lignin and arabinoxylan in the plant cell wall, with the ferulates ether-linked to lignin and ester-linked to the arabinoxylan [27]. Ammonolysis of the ferulate ester linkages to arabinoxylan side-chains is believed to be one major reaction occurring during the AFEX process [28]. These mechanisms may be opening up the cell wall ultrastructure more effectively at the higher ammonia loading, allowing the enzymes greater access to cellulose. Also, by increasing access to the substrate, the xylanase enzymes would have more potential xylan binding sites and therefore be less likely to bind competitively to the cellulose chains. This could explain the increase in glucose yields, with the addition of xylanase, for 1.5 (g NH_3 _g^-1 ^biomass).

These hypotheses are supported by the fact that the husk, the material with the lowest lignin content, while having the second-highest xylan content, is least affected by the combination of increased ammonia loading and xylanase addition. At 1.5 (g NH_3 _g^-1 ^biomass), the xylose yield only increases by 6.1% with the addition of xylanase to the hydrolysis cocktail. The late cob, which has a significantly higher lignin and xylan content than all of the other materials, experiences the largest impact on xylose yields due to the combination of increased ammonia loading and addition of xylanase - a 22.5% increase. The higher ammonia loading would cleave more linkages between the hemicellulose and lignin, solubilizing more oligomeric and monomeric xylose and, perhaps, some lignin as well. These exposed, solubilized sugars would be much easier to hydrolyze with the xylanase. It might be possible, given the very high xylan content of the cob, that more xylanase would be needed to achieve near complete monomeric xylose yields. As the xylanase loading was based on a percentage of the cellulase loading (and therefore the glucan content), and because the g glucan g^-1 ^xylan ratio for the cob (0.85 g glucan g^-1 ^xylan) is much lower than the other fractions (1.47 - 1.85 g glucan g^-1 ^xylan), the xylanase loading in terms of the xylan content (mg xylanase g^-1 ^xylan) is much lower for the cob fraction (Table 6). This may be one reason for the much lower xylose yield relative to the maximum theoretical xylose yield of the late cob fraction.

**Table 6:** Enzymatic hydrolysis xylanase loading in terms of xylan content of each fraction.

		Xylanase loading	Xylanase activity
Corn stover fraction		mg xylanase g^-1 ^xylan	OSX* g^-1 ^xylan
**Early**	**Leaves**	4.78	2891
	**Stem**	5.73	3456
**Late**	**Leaves**	5.03	3030
	**Stem**	4.97	2997
	**Husk**	4.57	2754
	**Cob**	2.64	1593

*Oat spelt xylan, based on activity numbers from Dien *et al*. (Ref [25])

### Statistical analysis

All AFEX parameters had significant impacts on sugar yields, except for temperature which had no significant effect on glucose yields. Based on least squares means analysis (data not shown), the temperature effect on xylose yield is likely due to a greater yield increase as the temperature is raised from 80° to 90°C rather than the decrease in yield when temperature is raised from 90° to 100°C. For the range of conditions tested, optimizing the ammonia loading, moisture content and residence time are more important for maximizing sugar yields from corn stover. However, this conclusion may change for a different range of temperatures and should not be extrapolated to other conditions.

Harvest date had a significant impact on xylose yields but not on glucose yields. This is largely due to the fact that the xylan content of the late fractions was greater than the xylan content of the early fractions, whereas the glucan content was not significantly different between harvests. The corn stover fractions tested had a significant effect on glucose yields but not on xylose yields. The late stem, husk, leaf and early stem fractions had no significant statistical difference in their glucan contents, so their relative recalcitrance, in terms of glucose yields, can be inferred from Figure 4. As the husk has the highest glucose yield, it can be considered the least recalcitrant, followed by the leaf and then the stem. Inferences cannot be made regarding the cob because its glucan content is statistically lower than the other three fractions. However, because the cob approaches theoretical glucan yields at optimal conditions while the stem does not (Figure 1), it may be less recalcitrant in terms of the conversion of glucan.

For interactive effects, only two were significant: harvest date × ammonia loading and corn stover fraction × xylanase addition. The harvest × ammonia interaction was significant for both glucose and xylose yields. The increase in ammonia loading from 1.0 to 1.5 (g NH_3 _g^-1 ^biomass) appears to have a greater effect on the late harvest than the early harvest, but this may be due to the lack of data for the early harvest cob. As the cob is the fraction most affected by the increase in ammonia loading, particularly for xylose yield, the lack of early cob data may lead to an apparent difference in effects that is not actually present between harvests. None of the other AFEX parameters show this relationship with either harvest date or corn stover fraction, which indicates that the same pretreatment conditions (moisture, temperature and residence time) can be used to maximize glucose release, regardless of the fractional composition of the corn stover or the harvest date.

The second significant interactive effect was for corn stover fraction × xylanase addition, but only for xylose yields. The main reason for this effect, as can be observed from Figure 4, is due to the cob fraction which was much more strongly affected by the addition of xylanase than all of the other fractions, whose responses were fairly similar. This conclusion is supported by the fact that when the data for the late cob was removed from the analysis, the fraction × xylanase interaction became non-significant (data not shown). Taken together, these results indicate that of all the corn stover components, the cob reacts more differently during enzymatic hydrolysis. As mentioned previously, the cob may require a much higher xylanase loading than the other fractions in order to release xylose remaining in the biomass or to convert the AFEX solubilized xylo-oligomers.

### Empirical modeling of harvest scenarios

As a result of the wide range of opinions on how much corn stover can be sustainably harvested and because the amount will likely change for a given field depending on environmental conditions and agricultural practices [4,6-8], we have modeled a number of corn stover harvest scenarios for both a liberal harvest estimate (70% of available corn stover) and a conservative harvest estimate (30% of available corn stover). The goal was to determine which combination of fractions provides the most benefit to the biorefinery in terms of sugar yields, and to determine the preferential order in which fractions should harvested from the field.

Crofcheck and Montross [17] found that the weighted sum of the glucose yields from individual pretreated fractions was not statistically different from the glucose yield from whole pretreated corn stover. This means that glucose yields for SHCS could be predicted using glucose yields from individual fractions. Our estimate of the late harvest dry matter distribution of corn stover (Figure 5), which was based on published data from four sources [15-17,22], is similar to standard estimates of corn stover dry matter distribution near corn harvest [29]. Corn stover dry matter yields, particularly of the husk and leaf, tend to decrease rapidly due to weathering over the course of the grain harvest season [15,16,21-24,30]. This estimate attempts to account for both the effects of the late harvest date as well as our inclusion of the leaf sheath with the leaf fraction instead of the stem fraction, as is often the case [15,16].

The estimated whole corn stover dry matter distribution was used to predict monomeric glucose and xylose yields from the three different harvest scenarios and the worst case scenario (where the least digestible fractions were harvested) for both a 70% (Table 4) and a 30% (Table 5) harvest of on-field corn stover using weighted averaging of individual fraction sugar yields. It is important to note that values given in these tables do not attempt to take into account the ability or inability to harvest the specific fractions or any losses due to inefficiencies in harvest, transport and storage of corn stover, which can be significant depending on the methods used.

A recent study found that the maximum amount of corn stover was available at grain physiological maturity (15.6 t ha^-1^) and steadily decreased over the harvest period to a minimum of 8.6 t ha^-1 ^[30]. As this value takes into account the late season of harvest, and because it is within the range of most estimates of corn stover yields reported in the published literature (7.8 - 8.8 t ha^-1^) [1,2,31], 8.6 t ha^-1 ^of available corn stover was chosen to estimate the total sugars that could be produced per hectare for the given harvest scenario. The standard value of 0.51 (theoretical g ethanol produced g^-1 ^sugar consumed) was used to determine the theoretical ethanol production from both a kilogram of SHCS and a hectare of harvested SHCS and does not take into account inefficiencies of fermentation.

Harvest scenario A, which selectively harvests the husk followed by the leaf, stem and, lastly, the cob, obtained the highest sugar and ethanol yields of all the scenarios and, as a result, was chosen as the optimal harvest scenario for AFEX-treated corn stover. Harvest scenario A was also preferable to scenarios B and C for a number of other reasons. First, optimizing the collection for maximum glucose yields is preferable because most current and relevant microbial strains selectively utilize hexoses over pentoses as a carbon source during ethanolic fermentation [12,32]. Second, harvest scenario A selectively leaves behind the more lignified fractions on the field which may prove more valuable for improving SOC levels due to the longer half-life of lignin compared to cellulose and hemicellulose [4,33]. Lastly, harvest scenario A seems to be the most feasible option from a technical viewpoint.

Selective harvesting of corn stover fractions will involve either returning the cob and/or husk to the field following the removal of the grain from the ear and/or raising the header on the combine to increase the stover cut height [2,34,35]. As a result of the association of the leaves with the stem, at higher cut heights it would be almost impossible to remove all of the leaves while leaving the entire stem behind. Taking these factors into consideration, of all of the scenarios, the most feasible from a technical aspect would be: scenario A (70% harvest), where all of the cob and a portion of the lower stem is returned to the field; and scenario C (30% harvest), where only the stover associated with the ear (husk and cob) is retained. Scenario A (30% harvest) could also be feasible if we replaced the percentage associated with the leaf material with a mixture of the upper-most portion of the corn plant (leaf and stem). This might be a reasonable option, because the upper portion of the stem tends to be more easily digestible than the lower portion of the stem and also has a higher sugar content than the leaf [26,34]. So, harvesting the upper portion of the corn plant could hypothetically give higher yields than harvesting the leaf alone. Unfortunately, this cannot be modeled because, for this study, only the entire, homogenized corn stem was tested.

Crofcheck and Montross [17] recommended, based on glucose yields from fractionated corn stover, a roughly 30% corn stover harvest scenario where the SHCS was composed of all of the available cobs and 74% of the leaves and husks, leaving the most recalcitrant stalks on the field. The difference between their optimal harvest scenario and ours is most probably due to their experimental methods for pretreatment and the subsequent analysis. Pretreatment of lignocellulosic biomass, using dilute sodium hydroxide, solubilizes much of the lignin and some of the hemicellulose into the liquid pretreatment stream [36,37]. It is therefore unlikely that glucan content of the pretreated corn stover corresponds to glucan content of the untreated corn stover. For similar pretreatment conditions of corn stover, Varga *et al*. found a 41.9% mass loss from the untreated dry corn stover to the pretreated solids and the composition of the pretreated material shifted in favour of a higher glucan content [37]. The cob has a significantly higher xylan and lignin content than the other fractions of the corn plant and, therefore, it is reasonable to assume that it will lose a greater proportion of its mass following dilute alkali pretreatment. As this mass loss was not taken into account [17], the amount of glucan that could be obtained on a mass basis from the untreated fractions was over-exaggerated, particularly from the xylan- and lignin-rich cob. If the mass loss had been taken into account, it is likely that their choice of optimal fractions for harvest would have been different. As AFEX is a dry-to-dry process with little mass loss during pretreatment, the glucan content of the pretreated material can be assumed to be the same as the glucan content of the untreated material [11]. It is feasible, because of differences in reaction chemistries, that other pretreatment methods would give different results for the selective harvest ratio of corn stover fractions compared to those for AFEX. However, because Crofcheck and Montross did not take into account the mass losses which occurred during their pretreatment and as we therefore do not know their sugar yields based on the untreated stover fractions, we cannot attribute the difference between our results and theirs to differences between the pretreatment methods. Rather the difference is likely due to errors in their analysis.

Shinners *et al*. [34] analysed the effect of cut height of corn stover (a harvest scenario that leaves a portion of the lower stem and leaves behind) on predicted ethanol yields and found that the amount of ethanol produced was only ~3% greater (L Mg^-1 ^DM) for the low cut compared to the high cut. If you were to assume that the amount of material harvested per hectare was constant, focusing only on the composition differences in the harvested material, this result would indicate that the fraction harvested has little impact on the theoretical ethanol production, which is similar to our results. However, when they analyzed their results based on the ethanol yield per hectare, the increase in total dry matter harvested with the lower cut height increased the predicted ethanol yield by 52% compared to the higher cut [34], which indicates that the amount of material harvested has a significant impact on theoretical ethanol yields and corresponds to our findings.

Based on these results, optimizing the fractions collected during harvest has a much smaller impact on potential yields than optimizing pretreatment and hydrolysis conditions, even if the worst case scenario occurs and the least digestible materials are preferentially harvested. However, the amount of stover harvested has the greatest impact on theoretical ethanol production per hectare. It will be very important, in terms of maximizing ethanol production, to develop methods to efficiently maximize harvest of corn stover, while still maintaining soil productivity and preventing erosion.

## Conclusion

Based on monomeric glucose and xylose yields, the optimal AFEX conditions, for all stover fractions (leaf, stem, husk and cob) regardless of harvest period, were found to be 1.5 (g NH3 g-1 biomass), 60% moisture content (dwb), 90°C and 5 min residence time; with enzyme loading during hydrolysis of 31.3 mg of cellulase (Spezyme^® ^CP, New York, USA), 41.3 mg of β-glucosidase (Novozyme^® ^188, Babsvaerd, Denmark) and 3.1 mg xylanase, g^-1 ^glucan. These conditions are different from those presented in previous analyses [9,10] largely due to the inclusion of xylanase in the hydrolysis cocktail. The addition of xylanase was necessary in order to achieve high xylose yields at moderate cellulase loadings and moderate AFEX conditions, particularly with respect to the more recalcitrant cob and stem fractions.

The optimal harvest scenario for the collection of SHCS would harvest the husk followed by the leaves, then the stem, and, lastly, the cob. This harvest scenario was independent of ammonia loading during AFEX pretreatment and maximized glucose and ethanol yield from SHCS. This scenario, combined with the optimal AFEX pretreatment conditions for SHCS, gave a theoretical ethanol yield of 2051 L ha^-1 ^for the 70% dry matter harvest and 912 L ha^-1 ^for the 30% dry matter harvest. Decreasing the stover collection from 70% to 30% dropped the ethanol yield by 852 - 1139 L ha^-1^, depending on harvest scenario and pretreatment conditions. Maximizing stover collection while protecting soil health will be the most important factor for maximizing ethanol yields from corn stover.

Optimizing the collection of corn stover fractions has little impact on the theoretical ethanol yield (29 - 141 L ha^-1^), especially compared to optimizing pretreatment and hydrolysis conditions (150-462 L ha^-1^). The dry matter distribution of collected corn stover fractions is generally much less important than the optimization of the ethanol production process. However, it is still something that needs to be taken into account because harvesting the worst fractions can decrease ethanol yields considerably, especially when a smaller percentage of the stover is collected.

Due to differences in pretreatment chemistries, the results for the optimal harvest of corn stover fractions may depend on the pretreatment method used. However, the differences between the optimal harvest scenarios presented here and those in Crofcheck and Montross [17] are confounded by the errors in their analysis and, therefore, the two pretreatment methods cannot be compared.

## Methods

### Harvest and milling

Corn stover, from a variety intended for grain production, was manually harvested from the Michigan State University Agronomy Center in East Lansing, Michigan, USA in September (early harvest) and November (late harvest) of 2006. The early and late stover harvests were separately hand-sorted into four individual fractions: stems, leaves with leaf sheaths, cobs and husks. The early husk and early cob fractions were not used because of spoilage of the material prior to use. All other fractions were air-dried, with stems split lengthwise in order to increase the drying rate. Fractions were then milled using a Fitzpatrick JT-6 Homoloid mill (Continental Process Systems, Inc, Westmont, Illinois, USA), with leaf, husk and cob fractions passing through a 4.763 mm (3/16 in) mesh screen and stem fractions passing through a 3.175 mm (1/8 in) mesh screen.

### Composition analysis

Biomass moisture content was determined using a moisture analyser (A&D, Model MF-50; California, USA). The composition of each corn stover fraction (ash, lignin, glucan and xylan content) was determined using the National Renewable Energy Laboratory (NREL, Colorado, USA) standard protocols [38]: ash analysis, (LAP 005); removal of extractives (LAP 010); and structural carbohydrates and lignin (LAP 002, 003, 004, 007, 019). The acid insoluble lignin analysis method was modified to use 47 mm, 0.22 μm pore-size, mixed-cellulose ester filter discs (Millipore Corp, Massachusetts, USA) during the filtration step instead of fritted crucibles. Due to problems with burning, these discs, with their filtered lignin residue, could not be dried in the vacuum oven and were therefore dried overnight in a desiccator prior to weighing. Soluble sugars could not be quantified after extraction due to difficulties in resolving distinct peaks using the high-performance liquid chromatography (HPLC) and were therefore not included in the composition.

### AFEX treatment

A small-scale benchtop reactor system, consisting of four separate 22 mL stainless steel (No. 316) reaction vessels, was used for the pretreatment process. Prior to its loading, the biomass was adjusted to the appropriate moisture content with deionized water, after which 3.0 g (dwb) of biomass was added to each reaction vessel. A metal screen was placed over the biomass inside each vessel, to prevent any escape of biomass during venting. The loaded reactor units were weighed and then attached to the reactor manifold and any air within the reactor vessels was then removed using a rotary vacuum pump. Liquid anhydrous ammonia was dispensed into the manifold via Swagelok screw valves (Swagelok Co, Ohio, USA) and then added to the reactor vessels. The reactors were weighed in order to determine the amount of ammonia added and they were then vented slightly to reach the appropriate ammonia loading. A heating mantle was used to raise the reactors to the desired temperature and maintain it for the set residence time. On completion of the residence time, the reactor pressure was explosively released via a stainless steel (No. 316) ball valve and the reactor was simultaneously cooled. The pretreated biomass was removed from the vessel and left in the fume hood overnight to allow the residual ammonia to evaporate.

### Enzymatic hydrolysis

NREL protocol (LAP 009) [38] was followed for the enzymatic hydrolysis of pretreated and untreated (control) samples. All samples were hydrolyzed in 20 mL screw-cap vials at 1% glucan loading and a total volume of 15 mL. Samples were adjusted to a pH of 4.8 by 1 M citrate buffer solution. Spezyme^® ^CP (Genencor Division of Danisco US, Inc, New York, USA) cellulase at 15 FPU g^-1 ^glucan (31.3 mg protein g^-1 ^glucan) and β-glucosidase (Novozyme^® ^188, Novozymes Corp) at 64 p-NPGU g^-1 ^glucan (41.3 mg protein g^-1 ^glucan) were added to each vial with a total protein content of 72.6 mg protein g^-1 ^glucan. In addition, certain samples were also hydrolyzed using xylanase (Multifect^® ^Xylanase, Genencor Division of Danisco US Inc) at 10% of total cellulase protein (1871 OSX (oat spelt xylan) g^-1 ^glucan or 3.1 mg protein g^-1 ^glucan), giving a total protein content of 75.7 mg protein g^-1 ^glucan. The data for the xylanase activity are based on the activity per mL provided by Dien *et al*. (2008) [31] and the activity, in terms of the xylan content of each sample, is included in Table 6. Enzyme loading throughout the paper is referred to in terms of protein loading, as opposed to activity, because of the probable relationship between protein and enzyme cost to the biorefinery [39]. Samples were placed in a New Brunswick Scientific (New Jersey, USA) incubator shaker and hydrolyzed at 50°C and 150 rpm for 72 h. The hydrolysates were sampled at 24 h and 72 h, following which samples were heated at 90°C for 15 min, cooled and centrifuged at 15 K for 5 min. The supernatant was filtered into HPLC shell vials using a 25 mm, 0.2 μm polyethersulfone syringe filter (Whatman Inc, New Jersey, USA) after which samples were stored at -20°C until further sugar analysis.

### Sugar analysis

An HPLC system was used to determine the sample monomeric glucose and xylose concentrations following enzymatic hydrolysis. The HPLC system consisted of a Waters (Massachusetts, USA) pump, auto-sampler and Waters 410 refractive index detector, equipped with a Bio-Rad (Hercules, California, USA) Aminex HPX-87P carbohydrate analysis column with attached deashing guard column. Degassed HPLC grade water was used as the mobile phase, at 0.6 mL/min, with the column temperature set at 85°C. Injection volume was 10 μL with a run time of 20 min per sample. Mixed sugar standards were used to quantify the amount of monomeric glucose and xylose in each hydrolysate sample. As there is no pretreatment liquid stream, all sugar yields are from the enzymatic hydrolysate and are reported in terms of the untreated dry biomass.

### Statistical analysis

Monomeric glucose and xylose yields following enzymatic hydrolysis were analysed using MANOVA in Minitab15 Statistical Software (2006 Minitab Inc, Pennsylvania, USA). The interactive effects plot which compares the harvest period and the stover fraction with each other, the four AFEX pretreatment parameters (moisture content, ammonia loading, temperature and residence time) and the xylanase addition was also constructed using Minitab.

### Empirical modeling of harvest scenarios

For this analysis, the sugar yields used were from the 72 h hydrolysis of AFEX-treated late corn stover. The option of an early harvest was not analyzed because of the lack of data for husk and cob fractions. Scenarios were analyzed with regard to the effect of increasing ammonia loading from 1.0 to 1.5 (g NH_3 _g^-1 ^biomass) and for the maximized sugar yield, either glucose or xylose. This gave four potential scenarios (1.0 + glucose, 1.5 + glucose, 1.0 + xylose and 1.5 + xylose). All other AFEX and hydrolysis conditions were held constant (60% dwb moisture, 90°C, 5 min residence time + 10% xylanase addition). As the glucose yields were consistently higher than the xylose yields, the harvest conditions used to obtain maximum glucose yields for all of the scenarios also corresponded with the maximum total sugar yields.

## Abbreviations

AFEX: ammonia fiber expansion; DM: dry matter; dwb: dry weight basis; HPLC: high-performance liquid chromatography; MANOVA: multivariate analysis of variance; SHCS: selectively harvested corn stover; SOC: soil organic carbon.

## Competing interests

The authors declare that they have no competing interests.

## Authors' contributions

RG designed and performed the experimental work, evaluated the results and wrote the manuscript. SC helped to conceive the study, evaluate the results and draft the manuscript. VB helped to conceive and coordinate the study and draft the manuscript. BD helped draft the manuscript. All authors read and approved the final manuscript.
